# Effectiveness of a novel bubble CPAP system for neonatal respiratory support at a referral hospital in the Philippines

**DOI:** 10.3389/fped.2023.1323178

**Published:** 2023-12-15

**Authors:** Paula Rauschendorf, Ghassan Bou Saba, Grace K. Meara, Navid Roodaki, Agustin Conde-Agudelo, Daisy Evangeline C. Garcia, Thomas F. Burke

**Affiliations:** ^1^ Vayu Global Health Foundation, Boston, MA, United States; ^2^ Department of Emergency Medicine, Massachusetts General Hospital, Boston, MA, United States; ^3^ Section of Neonatology, Department of Pediatrics, Ilocos Training and Regional Medical Center, San Fernando City, Philippines; ^4^ College of Medicine, Mariano Marcos State University, City of Batac, Philippines; ^5^ College of Medicine, University of Northern Philippines, Vigan City, Philippines; ^6^ Oxford Maternal and Perinatal Health Institute, Green Templeton College, University of Oxford, Oxford, United Kingdom; ^7^ Harvard Medical School, Boston, MA, United States; ^8^ Harvard T.H. Chan School of Public Health, Boston, MA, United States

**Keywords:** CPAP therapy, neonatal mortality, low-resource settings, respiratory support, preterm birth, neonatal intensive care unit

## Abstract

**Aim:**

To examine the impact of introducing and implementing the Vayu bubble continuous positive airway pressure (bCPAP) system on neonatal survival and neonatal respiratory outcomes in a neonatal intensive care unit (NICU) in the Philippines.

**Methods:**

We compared clinical outcomes of 1,024 neonates before to 979 neonates after introduction of Vayu bCPAP systems into a NICU. The primary outcome was survival to discharge. Adjusted odds ratios (aORs) with 95% confidence intervals (CIs) were calculated. Analyses were undertaken separately for the entire NICU population and for neonates who received any form of respiratory support.

**Results:**

The introduction of the Vayu bCPAP system was associated with (1) significant reductions in intubation (aOR: 0.75; 95% CI: 0.58–0.96) and in the use of nasal intermittent positive-pressure ventilation (NIPPV) (aOR: 0.69; 95% CI: 0.50–0.96) among the entire NICU population and (2) a significant increase in survival to discharge (aOR: 1.53; 95% CI: 1.09–2.17) and significant reductions in intubation (aOR: 0.52; 95% CI: 0.38–0.71), surfactant administration (aOR: 0.60; 95% CI: 0.40–0.89), NIPPV use (aOR: 0.52; 95% CI: 0.36–0.76), and a composite neonatal adverse outcome (aOR: 0.60; 95% CI: 0.42–0.84) among neonates who received any form of respiratory support.

**Conclusion:**

The use of the Vayu bCPAP system in a NICU in the Philippines resulted in significant improvement in neonatal respiratory outcomes.

## 1. Introduction

In 2021, 2.3 million infants worldwide died in their first month of life (1). Complications related to preterm birth are the leading cause of neonatal death around the world, most of which occur in low-resource settings (2–4). Respiratory distress, particularly respiratory distress syndrome (RDS), is the single most important cause of complications and death in preterm infants (5). Currently, continuous positive airway pressure (CPAP) therapy is recommended for the treatment of preterm infants (<37 weeks of gestation) with RDS and may be considered immediately after birth for very preterm infants (<32 weeks of gestation) with or without respiratory distress (6, 7).

Treatment of neonates with CPAP improves gas exchange, decreases the work of breathing, and can reduce mortality by as much as 66% (8). CPAP therapy also decreases the need for surfactant administration and mechanical ventilation and reduces the length of hospital stay and referrals to higher levels of care (8–10). In the November 2022 World Health Organization (WHO) *Recommendations for Care of the Preterm or Low-Birth-Weight Infant*, initiation of CPAP therapy was strongly recommended for neonates with respiratory distress (6, 7). The WHO has also recommended considering the use of bubble CPAP (bCPAP), a method that provides CPAP by using a column of water to generate pressure that is carried into the pulmonary system, for preterm infants for whom CPAP is warranted (6, 7). This recommendation was based on evidence of small-to-moderate decreased risk of pneumothorax, decreased bronchopulmonary dysplasia, and decreased failed treatment in trials involving preterm infants (6, 7).

Neonatal mortality has not decreased over the past five years in the Republic of the Philippines (11). High costs of commercially available CPAP devices, lack of consumables and compressed air for medical purposes, and the need for uninterrupted electricity are barriers to the provision of CPAP worldwide and throughout the Philippines (12). The novel Vayu bCPAP system has been designed specifically to overcome barriers to global access to CPAP. This system generates continuous pressure throughout the respiratory system and delivers blended, humidified, and filtered breathing gases at precise oxygen concentrations without the use of electricity (13). In early 2021, a total of 14 Vayu bCPAP systems were introduced and implemented in the neonatal intensive care unit (NICU) of the Ilocos Training and Regional Medical Center (ITRMC) in the Philippines. Details regarding the Vayu bCPAP system have been described previously elsewhere (13).

The objective of this study was to evaluate the impact of introducing and implementing Vayu bCPAP systems on neonatal survival and on the incidence of intubation, surfactant administration, and use of nasal intermittent positive-pressure ventilation (NIPPV) in the NICU at the ITRMC in the Philippines.

## 2. Methods

### 2.1. Study design

This was a baseline (admissions from March 1, 2020 to February 28, 2021) and intervention (admissions from March 1, 2021 to February 26, 2022) comparative study that evaluated the impact of introducing Vayu bCPAP systems for neonatal respiratory support into the NICU at ITRMC. Demographic and clinical characteristics and outcomes of neonates admitted to the ITRMC NICU during the two study periods were compared. The entire NICU population and a subgroup of neonates who received any form of respiratory support (intubation, NIPPV, CPAP therapy, low-flow oxygen, or low-flow medical compressed air) were evaluated separately.

### 2.2. Study setting

ITRMC is a public regional referral hospital in San Fernando City, La Union, the Philippines. The pediatric department has a level III NICU with 25 beds. This NICU can provide 40 beds when needed. The equipment in the NICU includes radiant warmers, incubators, and monitoring equipment. Support for kangaroo mother care (i.e., skin-to-skin contact between the mother and infant) and breast-feeding is available 24 h daily. Surfactant therapy is readily available, and the cost is covered by the Philippines Health Insurance Corporation. The average ratio of nurses to neonates in the NICU is one to 10.

Before the implementation of the Vayu bCPAP systems, three CareFusion synchronized inspiratory positive airway pressure (SiPAP) devices, two Fisher & Paykel CPAP machines, and one Dräger CPAP device were available for use in the ITRMC NICU. Ventilator-driven CPAP therapy was also available. The reuse of consumables for the Fisher & Paykel CPAP devices began three quarters of the way through the baseline period (December 2020), and Dräger and CareFusion consumables became unavailable due to a lack of affordability. Since early in the intervention period, 14 Vayu bCPAP systems, two Fisher & Paykel CPAP devices, and mechanical ventilators in CPAP mode were available for provision of CPAP in the ITRMC NICU. Throughout both the baseline and the intervention periods, only two staff members were able to operate the CareFusion SiPAP systems, and there was no set number of available mechanical ventilators because the NICU shares the 30 mechanical ventilators in the hospital (Mindray, Puritan Bennet, and Hamilton-G5 ventilators) with all hospital departments.

Infants admitted to the ITRMC NICU vary from the critically ill to those who are premature, have very low birth weight (LBW), or both. Premature infants are discharged from the NICU only once they reach 1.2–1.5 kg. Guidelines for initiation of CPAP therapy, NIPPV use, and intubation did not change between the baseline and intervention periods. Prophylactic application of CPAP therapy was not practiced at ITRMC at any point during the study. Surfactant was administered to neonates whose condition did not improve with a fraction of inspired oxygen (FiO_2_) of 30% while receiving CPAP and to those who required intubation. Apneic infants and those in whom CPAP or NIPPV therapy failed were intubated.

### 2.3. Intervention

#### 2.3.1. Introduction of Vayu bCPAP systems

At the beginning of March 2021, four Vayu bCPAP systems were introduced into the ITRMC NICU. In April 2021, 10 more Vayu bCPAP systems were added for a total of 14 devices available during the intervention period.

#### 2.3.2. Training

Initial training in the use of the Vayu bCPAP system was performed virtually on Zoom by the Vayu Global Health Foundation implementation team. The training was conducted in English, translations were not needed. The training covered device assembly, application, troubleshooting, and reprocessing. Attendees of the virtual training session assumed the roles of master trainers in charge of cascade training in the NICU. They subsequently transferred knowledge and skills to the NICU staff via one-to-one mentorship during shifts. A link to an online certification course on the Vayu bCPAP system was provided but participation was voluntary.

### 2.4. Data collection

#### 2.4.1. Demographic and clinical characteristics of the neonates

Data on all neonates admitted to the ITRMC NICU during the baseline period were collected retrospectively from patient files. Data on all neonates admitted during the intervention period were collected prospectively. Sex, birth weight, gestational age at birth, delivery method (vaginal or cesarean delivery), primary diagnosis, and vital signs [heart rate, respiratory rate, temperature, and oxygen saturation as measured by pulse oximetry (SpO_2_)] were obtained for every neonate admitted to the NICU. The use of CPAP and other respiratory support therapies was recorded. A patient was recorded as a primary user of CPAP if their first form of advanced respiratory support after being admitted to the NICU was CPAP therapy. The primary diagnosis was recorded and tabulated for each NICU admission.

#### 2.4.2. Outcomes

The primary outcome was survival to discharge. Secondary outcomes included intubation, surfactant administration, NIPPV use, length of NICU stay, and a composite neonatal adverse outcome defined as the occurrence of any of the following: death, intubation, surfactant use, or NIPPV use.

### 2.5. Statistical analysis

Analyses were conducted in the two groups of interest: the entire NICU population and neonates who received any form of respiratory support. Between-group differences in demographic and clinical characteristics were assessed with univariate analyses. Categorical data were presented as total numbers and percentages, and continuous variables were expressed as means (±SD). Categorical data were analyzed with the use of chi-square or Fisher's exact testing, and continuous data were analyzed with the use of a *t*-test for parametric data and the Wilcoxon rank-sum test for nonparametric data. Two-sided *P* values of less than 0.05 were considered to be statistically significant. Unadjusted and adjusted odds ratios (ORs) with 95% confidence intervals (CIs) were calculated as the measure of association between the exposure (introduction of the Vayu bCPAP system) and the outcomes. Differences in outcomes between the two study periods were determined with the use of logistic-regression models for binary outcomes and linear regression models for continuous outcomes. In these models, assignment to the baseline and intervention periods was the independent variable and the outcome was the dependent variable.

To account for the potential effect of confounders, analyses were adjusted for demographic and clinical characteristics that were observed to have statistically significant differences between the two study periods (using a *P* threshold of <0.10 for inclusion in the adjustment). Primary diagnoses were not included in the logistic-regression models because senior providers at the trial site indicated that many of the diagnoses were interchangeable. Since neonates were kept in the NICU until they met specific weight-gain criteria, irrespective of their clinical condition, lengths of stay were recorded but were not analyzed further. Analyses were conducted with the use of R software, version 4.2.1 (R Foundation).

### 2.6. Ethical considerations

The study was approved by the technical and ethical review board of ITRMC (ITRMC REC-2022-04).

## 3. Results

### 3.1. Entire NICU population

Between March 2020 and February 2022, a total of 2,003 neonates were admitted to the NICU at ITRMC: 1,024 during the baseline period and 979 during the intervention period. The two groups were similar in terms of sex, gestational age at birth, birth weight, incidence of LBW, and vital signs on admission (Table 1). The proportion of infants who were delivered by cesarean section was statistically significantly higher in the intervention period than in the baseline period. Of the 2,003 neonates admitted to the NICU, 639 (31.9%) received some form of respiratory support—310 (30.3%) in the baseline group and 329 (33.6%) in the intervention group. CPAP therapy was the primary respiratory intervention for 24 neonates (2.3%) in the baseline group and 129 infants (13.2%) in the intervention group (*P* < 0.001), 119 (92.2%) of whom were treated with Vayu bCPAP systems.

**Table 1:** Demographic and clinical characteristics of infants admitted to the NICU, before and after introduction of the Vayu bCPAP system^a^.

Characteristic	Baseline period (*N* = 1,024)	Intervention period (*N* = 979)	*P* value
Gestational age at birth—weeks	36.7 ± 2.6	36.8 ± 2.9	0.73
Birth weight—kg	2.5 ± 0.7	2.5 ± 0.7	0.70
Birth weight <2,500 g—no. (%)	583 (56.9)	534 (54.5)	0.28
Male sex—no. (%)	531 (51.9)	527 (53.8)	0.38
Delivery method—no. (%)			<0.001
Vaginal	767 (74.9)	657 (67.1)	
Cesarean	257 (25.1)	322 (32.9)	
Vital signs on admission to the NICU			
Heart rate—beats/min	157.1 ± 9.6	158.1 ± 8.1	0.02
Breathing rate—breaths/min	62.7 ± 5.1	62.7 ± 4.8	0.78
Temperature—°C	37.0 ± 0.3	37.0 ± 0.3	0.90
SpO_2_—%	95.1 ± 3.0	94.6 ± 2.2	<0.001
Primary CPAP use—no. (%)^b^	24 (2.3)	129 (13.2)	<0.001
Admission diagnosis—no. (%)			
Small for gestational age	357 (34.9)	296 (30.2)	
RDS, pneumonia, or both	286 (27.9)	232 (23.7)	
Mother had Covid-19 when infant was born	0 (0)	157 (16.0)	
Sepsis	97 (9.5)	77 (7.9)	
Prematurity	51 (5.0)	38 (3.9)	
Jaundice	66 (6.4)	32 (3.3)	
Mother had diabetes when infant was born	47 (4.6)	24 (2.5)	
Large for gestational age	26 (2.5)	27 (2.8)	
Mother had hepatitis when infant was born	26 (2.5)	22 (2.2)	
Birth asphyxia	12 (1.2)	7 (0.7)	
Other	56 (5.5)	67 (6.8)	

^a^ Plus-minus values are means ± SD. The abbreviation bCPAP denotes bubble continuous positive airway pressure, Covid-19 coronavirus disease 2019, CPAP continuous positive airway pressure, NICU neonatal intensive care unit, RDS respiratory distress syndrome, and SpO_2_ oxygen saturation as measured by pulse oximetry.

^b^ Primary CPAP use involves use of CPAP as the primary mode of respiratory support.

The introduction and implementation of the Vayu bCPAP system in the entire NICU population was associated with a significant (25%) reduction in the odds of intubation (adjusted OR: 0.75; 95% CI: 0.58–0.96; *P* = 0.02) and a significant (31%) reduction in the odds of the use of NIPPV (adjusted OR: 0.69: 95% CI: 0.50–0.96; *P* = 0.026) (Table 2). In the overall NICU population there were no statistically significant differences between the baseline and intervention groups with respect to survival to discharge, surfactant use, length of NICU stay, and the composite neonatal adverse outcome.

**Table 2:** Impact of Introduction of the Vayu bCPAP system on the entire NICU population^a^.

Outcome	Baseline period (*N* = 1,024)^b^	Intervention period (*N* = 979)	Crude OR or Mean difference (95% CI)	*P* value	Adjusted OR or Mean difference (95% CI)	*P* value
Survival to discharge—no. (%)	902 (88.1)	880 (89.9)	1.20 (0.91 to 1.59)	0.20	1.21 (0.91 to 1.56)	0.19
Surfactant treatment—no. (%)	106 (10.4)	98 (10.0)	0.96 (0.72 to 1.29)	0.80	0.92 (0.69 to 1.23)	0.56
Intubation—no. (%)	160 (15.6)	120 (12.3)	0.75 (0.59 to 0.97)	0.03	0.75 (0.58 to 0.96)	0.02
NICU stay—days	6.9 ± 7.0	6.9 ± 8.2	0.03 (−0.53 to 0.70)	0.94	−0.03 (−0.69 to 0.64)	0.94
NIPPV use—no. (%)	96 (9.4)	69 (7.0)	0.73 (0.53 to 1.01)	0.06	0.69 (0.50 to 0.96)	0.03
Composite neonatal adverse outcome—no. (%)^c^	218 (21.3)	208 (21.2)	0.97 (0.78 to 1.20)	0.76	0.94 (0.76 to 1.17)	0.57

^a^ Plus-minus values are means ± SD. NIPPV denotes nasal intermittent positive-pressure ventilation, and OR odds ratio.

^b^ The group evaluated in the baseline period was the reference group.

^c^ The composite neonatal adverse outcome was defined as the occurrence of any of the following events: death, intubation, surfactant use, or NIPPV use.

### 3.2. Neonates receiving any form of respiratory support

A total of 310 neonates received respiratory support of any kind (: intubation, NIPPV, CPAP therapy, low-flow oxygen, low-flow compressed air, or more than one of these types of therapy) in the baseline period as compared with 329 in the intervention group (Table 3). CPAP therapy was the primary respiratory-support intervention for 24 of these neonates (7.7%) in the baseline group and 129 of those (39.2%) in the intervention group (*P* < 0.001). Among the neonates who received respiratory support, there were no significant differences between the two groups with respect to gestational age at birth, birth weight, sex, incidence of LBW, vital signs on admission, and delivery method.

**Table 3:** Demographic and clinical characteristics of infants receiving Any form of respiratory support in the NICU before and after Introduction of the Vayu bCPAP system^a^.

Characteristic	Baseline period (*N* = 310)	Intervention period (*N* = 329)	*P* value
Gestational age at birth—weeks	35.3 ± 3.7	35.3 ± 3.3	0.90
Birth weight—kg	2.3 ± 0.8	2.2 ± 0.8	0.17
Birth weight <2,500 g—no. (%)	167 (53.9)	200 (60.8)	0.08
Male sex—no. (%)	165 (53.2)	187 (56.8)	0.36
Delivery method—no. (%)			0.06
Vaginal	219 (70.6)	208 (63.2)	
Cesarean	91 (29.4)	121 (36.8)	
Vital signs at admission to the NICU			
Heart rate—beats/min	157 ± 9.5	158 ± 7.9	0.16
Breathing rate—breaths/min	62.9 ± 5.9	63.0 ± 4.9	0.74
Temperature—°C	37.0 ± 0.3	37.0 ± 0.3	0.46
SpO_2_—%	94.3 ± 4.0	94.5 ± 2.4	0.56
Primary CPAP use—no. (%)	24 (7.7)	129 (39.2)	<0.001
Admission diagnosis			
RDS	97 (31.3)	112 (34.0)	
Pneumonia	92 (29.7)	101 (30.7)	
Sepsis	78 (25.2)	61 (18.5)	
Birth asphyxia	12 (3.9)	5 (1.5)	
Other	31 (10.0)	50 (15.2)	

^a^ Plus-minus values are means ± SD.

Among infants who received any form of respiratory support, the use of a Vayu bCPAP system was associated with a significant (53%) increase in the odds of survival to discharge (adjusted OR: 1.53; 95% CI: 1.09–2.17, *P* = 0.01), a significant (48%) decrease in the odds of intubation (adjusted OR: 0.52; 95% CI: 0.38–0.71, *P* < 0.001) and NIPPV use (adjusted OR: 0.52; 95% CI: 0.36–0.76, *P* < 0.001), and a significant (40%) decrease in the odds of surfactant administration (adjusted OR: 0.60; 95% CI: 0.40–0.89, *P* = 0.011) and the composite neonatal adverse outcome (adjusted OR: 0.60; 95% CI: 0.42–0.84, *P* = 0.003) (Table 4). The mean length of NICU stay was similar in the two study groups.

**Table 4:** Impact of Introduction of the Vayu bCPAP system on infants receiving Any form of respiratory support^a^.

Outcome	Baseline period (*N* = 310)^b^	Intervention period (*N* = 329)	Crude OR or Mean difference (95% CI)	*P* value	Adjusted OR or Mean difference (95% CI)	*P* value
Survival to discharge—no. (%)	203 (65.5)	245 (74.5)	1.54 (1.09 to 2.16)	0.01	1.53 (1.09 to 2.17)	0.01
Surfactant treatment—no. (%)	106 (34.2)	98 (29.8)	0.82 (0.59 to 1.14)	0.23	0.60 (0.40 to 0.89)	0.01
Intubation—no. (%)	160 (51.6)	120 (36.5)	0.54 (0.39 to 0.74)	<0.001	0.52 (0.38 to 0.71)	<0.001
NICU stay—days	13.6 ± 8.8	13.5 ± 11.1	−0.075 (−1.63 to 1.48)	0.93	−0.18 (to 1.74 to 1.39)	0.82
NIPPV use—no. (%)	96 (31.0)	69 (21.0)	0.59 (0.41 to 0.85)	0.004	0.52 (0.36 to 0.76)	<0.001
Composite neonatal adverse outcome—no. (%)^c^	203 (65.5)	188 (57.1)	0.73 (0.51 to 0.97)	0.03	0.60 (0.42 to 0.84)	0.003

^a^ Plus–minus values are means ± SD.

^b^ The group evaluated in the baseline period was the reference group.

^c^ The composite neonatal adverse outcome was the occurrence of any of the following events: death at discharge, intubation, surfactant use, and NIPPV use.

## 4. Discussion

This two-year study compared outcomes before and after introduction and implementation of Vayu bCPAP systems in the NICU at a public regional referral hospital in the Philippines. We found that the use of this system was associated with a significant reduction in intubation and NIPPV among the entire NICU population and a significant increase in survival to discharge and a significant decrease in intubation, surfactant administration, NIPPV use, and the composite neonatal adverse outcome among the subgroup of infants who received any form of respiratory support. The use of CPAP in the NICU was 5.6 times higher after introduction of the Vayu bCPAP system than in the use in the baseline period.

The increased use of CPAP in the intervention period was associated with a simultaneous decrease in intubation and NIPPV use. Although several options for CPAP were theoretically available before the introduction of Vayu systems, as in most hospitals throughout the world, the available medical devices listed in the inventory were often not consistent with clinical availability (14). The Vayu bCPAP system was specifically engineered to overcome common barriers to CPAP use such as a lack of consumables, bioengineering support, and medical air and the need for uninterrupted electricity, among other critical features (13). In the ITRMC NICU, there were no changes to staffing or clinical guidelines between the two study periods, and there was no instruction to use CPAP with greater frequency. The introduction of Vayu bCPAP systems appeared to directly cause a major shift from the use of invasive and noninvasive positive-pressure ventilation to CPAP.

In the overall NICU population, less than one third of the neonates (31.9%) over both study periods had respiratory distress for which any form of respiratory treatment was received. Although there were significant reductions in the odds of intubation and NIPPV use (25% and 31%, respectively) after the introduction of bCPAP systems, given that the majority of neonates in the overall NICU population (68.1%) had nonrespiratory clinical conditions, it is not surprising that there were no statistically significant differences in survival to discharge or surfactant use.

To better evaluate the impact of introduction of Vayu bCPAP systems in neonates who could potentially benefit from the intervention, we conducted an analysis involving the population of newborns who had received any form of respiratory support in both the baseline and intervention groups. Since there were no major differences between these two groups with respect to patient clinical characteristics or the environments ecosystems in which they received care, the 53% (*P* = 0.01) improved survival could be attributed to the sharp increase in the use of CPAP, and the simultaneous 48% decrease in the odds of both intubation and NIPPV use may have been afforded by the introduction of the bCPAP system.

An additional effect of the introduction of the Vayu system was the reduction in surfactant administration. In the population of neonates who had received any form of respiratory support, the odds of surfactant administration decreased by 40% (*P* = 0.01) because neonates no longer had hypoxia at the frequency seen previously. The finding that fewer newborns met criteria for surfactant administration after the intervention suggests that there were considerable improvements in the clinical conditions of the newborns after introduction of Vayu systems. It is notable that the unexpected extra surfactant vials were redistributed to other hospitals in the surrounding region.

CPAP therapy has been shown to increase survival and decrease types of disability such as bronchopulmonary dysplasia in highly-resourced hospitals; however, studies conducted in low-resource settings have had mixed outcomes (15–19). The introduction of a device alone cannot improve outcomes. A medical device must be appropriate for a setting; technical support and the supply chain must be responsive to the health system's needs; interval training and mentorship around the role of the device in quality care are critical; and there must be integration of the devices into the overall health care delivery system (20).

The major strengths of this study are the rigorous methodology used in its conduct, the large number of participants relative to similar studies, the similarity in baseline characteristics in the baseline and intervention groups, and the limitation of the overall study time frame to 2 years, which decreased potential confounding factors of practice variability that may have affected patient outcomes.

Our study has some potential limitations. The outcomes in this before-and-after study may have been influenced by temporal confounders that were not measured, although the clinical setting, staffing, guidelines, and clinical characteristics of the two study groups were similar. In addition, the retrospective collection of data on the baseline group came with the inherent potential for lack of standardization in data recording. The coronavirus disease 2019 (Covid-19) pandemic influenced part of the intervention time period. The three relevant issues during the pandemic were that babies of Covid-19–positive mothers went to a separate unit and their data were included in the NICU database, health care staffing was often critically short, and no new programs were instituted other than the Vayu bCPAP system. The additional data on asymptomatic babies in the NICU database were removed in accordance with the study design in the analysis of the populations that received respiratory support, and the other two Covid-19-related issues contributed to lower or no change in the quality of clinical care. None of the Covid-19-related differences would have favored the respiratory-support intervention group. Finally, introduction of medical devices such as the Vayu bCPAP system is highly dependent on the clinical environment. Therefore, the findings from this study may not be generalizable to other settings.

Future research efforts should focus on implementation of the Vayu bCPAP system into settings where neonatal outcomes may be optimized. For example, early application in labor and delivery, use in transport, integration into lower-level facilities, and use during kangaroo mother care are all potential opportunities for quality care, but each must be evaluated for its contribution to outcomes.

In summary, introduction and implementation of the Vayu bCPAP system into the NICU of a regional referral center in the Philippines was associated with significant reductions in intubation and NIPPV use in the entire NICU population, as well as improved survival and a decreased incidence of intubation, NIPPV use, and surfactant administration among neonates who required any form of respiratory support.

## Data Availability

The raw data supporting the conclusions of this article will be made available by the authors, without undue reservation.
